# The Differences of Quantitative Flow Ratio in Coronary Artery Stenosis with or without Atrial Fibrillation

**DOI:** 10.1155/2023/7278343

**Published:** 2023-10-13

**Authors:** Wenbin Lu, Xiaoguo Zhang, Gaoliang Yan, Genshan Ma

**Affiliations:** Department of Cardiology, ZhongDa Hospital Affiliated with Southeast University, China

## Abstract

Quantitative flow ratio (QFR) is a new method for the assessment of the extent of coronary artery stenosis. But it may be obscured by the cardiac remodeling and abnormal blood flow of the coronary artery when encountering atrial fibrillation (AF). The present study aimed to examine the impact of these changed structures and blood flow of coronary arteries on QFR results in AF patients. *Methods and Results*. We evaluated QFR in 223 patients (112 patients with AF; 111 non-AF patients served as controls) who had undergone percutaneous coronary intervention (PCI) due to severe stenoses in coronary arteries. QFR of the target coronary was determined according to the flow rate of the contrast agent. Results showed that AF patients had significantly higher QFR values than control (0.792 ± 0.118 vs. 0.685 ± 0.167, *p* < 0.001). We further analyzed local QFR around the stenoses (0.858 ± 0.304 vs. 0.756 ± 0.146, *p*=0.002), residual QFR (0.958 ± 0.055 vs. 0.929 ± 0.093, *p*=0.005), and index QFR (0.807 ± 0.108 vs. 0.713 ± 0.152, *p* < 0.001) in these two groups of patients with and without AF. Further analysis revealed that QFR in AF patients was negatively correlated with coronary flow velocity (*R* = −0.22, *p*=0.02) and area of stenosis (*R* = −0.70, *p* < 0.001) but positively correlated with the minimum lumen area (MLA) (*R* = 0.47, *p* < 0.001). *Conclusion*. AF patients with coronary artery stenosis have higher QFR values, which are associated with decreased blood flow velocity, smaller stenosis, and larger MLA in AF patients upon cardiac remodeling.

## 1. Introduction

Fractional flow reserve (FFR) is considered the gold standard for the diagnosis of intracoronary insufficiency significance when stenosis is present. However, the invasiveness and complexity of operations, the side effects of intraoperative medication (such as adenosine or adenosine triphosphate, ATP), and the high cost of pressure guide wires (especially in developing countries) greatly limit the clinical applications of FFR.

Image-based, noninvasive QFR computing technology has emerged as an important technology in the interventional cardiovascular field in catheterization laboratories [1]. Clinical investigation has shown that QFR simplified the FFR testing process without employing an invasive pressure guide wire and achieved higher diagnostic accuracy without the usage of vasodilator drugs for microcirculation. The diagnostic sensitivity and specificity of QFR are significantly better than quantitative coronary angiography [2]. QFR results suggested that patients with lesions of QFR values less than 0.80 had a higher risk of adverse events. In addition, QFR has been included in expert consensus concerning acute myocardial infarction intervention in several countries [3, 4]. Therefore, QFR is a new tool of providing guidance for clinicians to formulate interventional treatment strategies in the catheterization room.

However, QFR still has limitations in evaluating the functional significance of certain coronary stenoses, and these limitations may cause unnecessary myocardial revascularization, for example, endothelial dysfunction caused by atherosclerotic disease or changes of blood flow in AF patients [5, 6]. As is well known that AF is always accompanied by irreversible cardiac remodeling including atrial and ventricular. At first, the enlargement and remodeling of the left atrium were dominant, and then the cardiac remodeling progressed with the enlargement of both the left and right atria as well as the ventricles. In this process, on the one hand, the coronary arteries located on the surface of the heart become distorted and deformed, resulting in abnormal coronary blood flow; on the other hand, the blood flow status of the atrium and ventricle also varied significantly. In the present study, we aimed to investigate the impact of the changes of blood flow and anatomical structure induced by cardiac remodeling on QFR results in AF patients.

## 2. Methods

### 2.1. Study Population

This is a retrospective study. Patients who were involved were less than or equal to 80 years old and were admitted to the Cardiac Center of Affiliated Zhongda Hospital, Southeast University, China. All patients were implanted with drug-eluting stents in coronary arteries from the year of 2014 to 2019 (the fact is that both groups of patients were with severe stenosis and had undergone interventional therapy assessed by QCA at least). For the homogeneity and uniformity between the groups, the heart rate of AF patients was effectively controlled by taking different doses of *β*-receptor inhibitors, and parts of the patients also received the potassium channel inhibitor amiodarone to control their heart rate. Inclusion criteria were as follows: (1) men or nonpregnant women ≥18 and ≤80 years of age; (2) AF patients with a CHA_2_DS_2_-VASc score ≥2 who received coronary stent implantation; and (3) non-AF patients who received coronary stent implantation. This program was approved by the Ethical Committee of the Affiliated Zhongda Hospital, Southeast University, China. All of the patients provided written informed consent. Subjects showing any of the following exclusion criteria were excluded from this study: >80 years old or <18 years old; estimated glomerular filtration rate (eGFR) of <30 mL/(minute∙1.73 m^2^); hemodynamic or electrical instability (including shock); and a platelet count of less than 90 × 10^9^/L.

All the AF group patients enrolled in the trial with at least one 12-lead electrocardiogram (*n* = 93) or a 24 hour Holter electrocardiogram (*n* = 19), and both have been taking oral anticoagulants (warfarin, etc) for at least 3 weeks. Based on the AF history of these patients [7], AF patients in this investigation involved paroxysmal AF (*n* = 27), persistent AF (*n* = 40), long-term persistent AF (*n* = 19), and permanent AF (*n* = 26). None of the above AF patients had undergone catheter radiofrequency ablation or balloon cryoablation.

### 2.2. Clinical Data Collection

**Figure 1:**
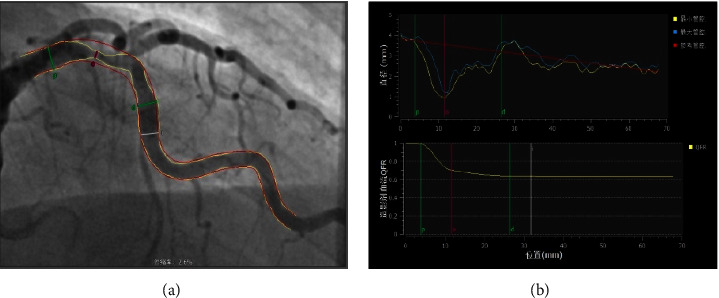
Schematic diagram of QFR measurement. (a) Different sites of coronary artery lesions were selected as pressure detection points. (b) The pressure values and overall curves of different sites of coronary artery lesions. The QFR was determined by Pd/Pa *∗* 100%.

Researchers interviewed patients and collected their medical histories from the medical charts. Basic characteristics of patients were acquired from clinical or biochemical tests, which included a history of cardiovascular or cerebrovascular diseases, smoking, drug intake, and blood pressure. The QFR in the whole target coronary, the local QFR around the stenoses, the residual QFR after stent implantation, and the index QFR after 3D reconstruction (Figure 1) were calculated using the Angio Plus system (Pulse Medical Imaging Technology Co., China) by an independent committee who were unaware of treatment allocation adjudicated and verified all required QFR-related values.

### 2.3. Definitions of Different Types of QFR

**Figure 2:**
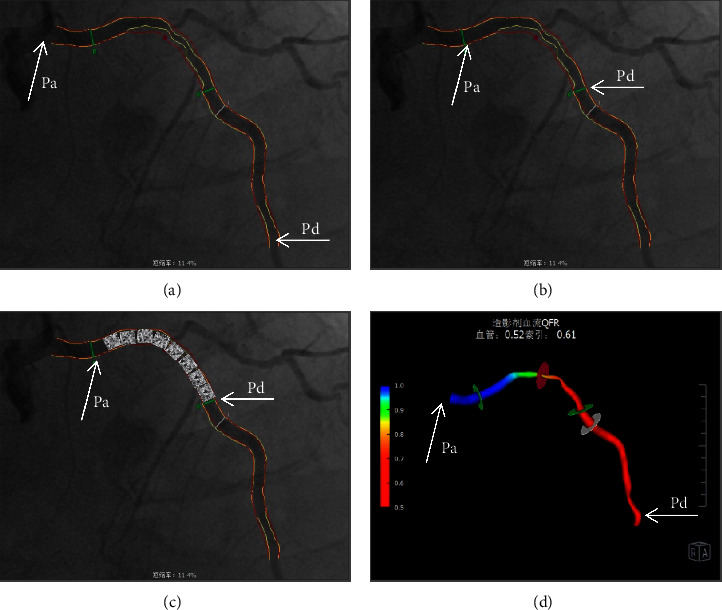
Different QFR types. (a) QFR of the whole target coronary artery. (b) Local QFR around the stenoses. (c) Residual QFR after stent implantation. (d) Index QFR after 3D reconstruction.

QFR of the target coronary artery is defined as the ratio of pressure at the farthest end of the target vessel to the pressure at the beginning of the coronary artery in the aortic sinus, and it is referred to as Pd/Pa (Figure 2(a)). Local QFR around the stenoses is the ratio of pressure at the distal lesion of the target vessel to the pressure at the proximal lesion, and it is referred to as Pd/Pa (Figure 2(b)). Residual QFR is the ratio of distal lesion pressure to proximal lesion pressure after stent implantation in target vessels, and it is referred to as Pd/Pa (Figure 2(c)). Index QFR is the ratio of pressure at the distal lesion to the pressure at the proximal lesion after 3D reconstruction of target vessels, and it is referred to as Pd/Pa (Figure 2(d)).

### 2.4. Statistical Analysis

Data management and statistical analyses were performed using SAS software version 9.1 (SAS Institute, USA). *p* ≤ 0.05 was considered statistically significant. Data are expressed as the mean ± standard deviation. Intergroup comparisons of continuous variables were performed using Student's *t*-test. Categorical variables were compared using the *χ*^2^ test.

## 3. Results

### 3.1. Patient Characteristics

A total of 223 patients in our cardiac center were enrolled, including 112 patients with AF and 111 patients without AF served as controls. The heart rates in the two groups of patients were comparable at the time of PCI (*p*=0.1318). The LvEF (%) of AF patients was slightly worse than that of the control group (59.65 ± 11.48 vs. 65.83 ± 5.78, *p* < 0.001); however, further analysis revealed no significant correlation between LvEF and QFR value (Supplementary Figure 1). The mean age of AF patients and the control group was 70.28 and 68.22 years old, respectively. Overall, 66.61% of patients in the AF group and 57.14% of patients in the control group were male patients. Clinical comorbidities between the two groups, including histories of hypertension, diabetes, and stroke/TIA, as well as the New York Heart Association classification grading of cardiac function and eGFR are shown in Table 1. Baseline procedural characteristics, including PCI-related vessels and periprocedural treatment, were all comparable (Table 2).

### 3.2. AF Patients Showed Higher QFR Results

**Figure 3:**
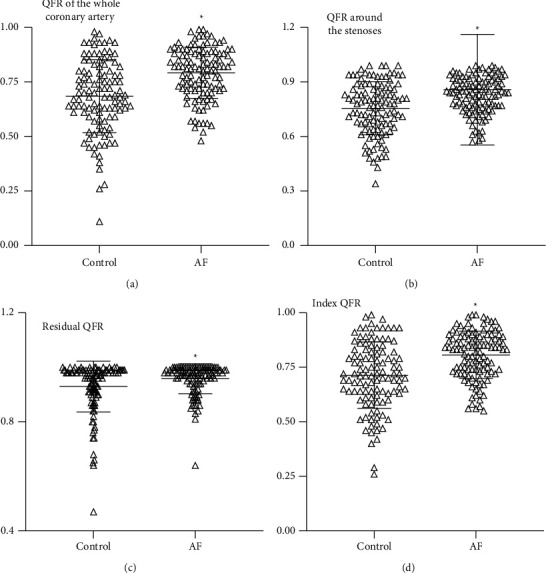
The QFR in AF patients and control patients. The QFR of the whole target coronary artery, local QFR, residual QFR, and index QFR were all higher in AF patients compared to non-AF patients.

There was a higher QFR of the whole diseased coronary artery in AF patients than in the control group (0.792 ± 0.118 vs. 0.685 ± 0.167, *p* < 0.001) (Figure 3). As is well known that patients with acute coronary syndrome (ACS), especially non-ST-segment elevation myocardial infarction (NSTEMI), are more likely to have microcirculatory disorders, to avoid the impact of microcirculation disorders on QFR results, we excluded ACS patients (17 ACS in the AF group and 14 ACS in the control group) and still found significant differences in QFR values between the two groups (0.814 ± 0.104 vs.0.705 ± 0.162, *p* < 0.001) (Supplementary Figure 2). Consistent with the results of the whole diseased coronary artery, local QFR around the stenoses (0.858 ± 0.304 vs. 0.756 ± 0.146, *p*=0.002), residual QFR (0.958 ± 0.055 vs. 0.929 ± 0.093, *p*=0.005), and index QFR (0.807 ± 0.108 vs. 0.713 ± 0.152, *p* < 0.001) were all higher in AF patients than controls. These consequences supported the hypothesis deduced from FFR measurement [8, 9].

### 3.3. AF Patients Showed Higher QFR Results in Corresponding Coronary Arteries

**Figure 4:**
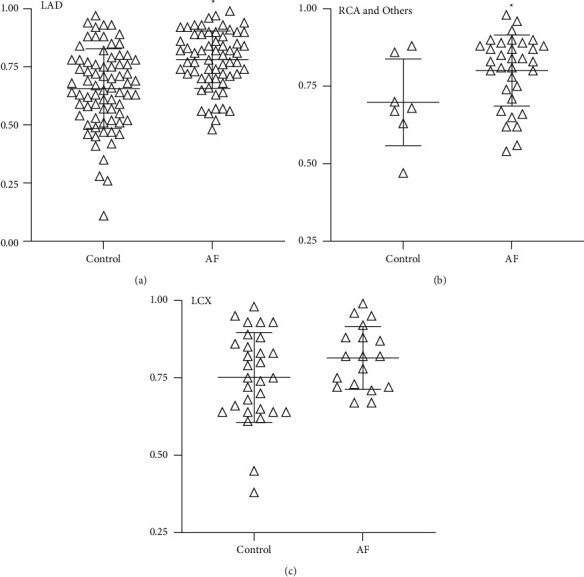
QFR results of certain coronary arteries in the AF and control groups. Higher QFR ratios were determined in the LAD (*p* < 0.001), RCA, and “others” (*p*=0.045) compared to the control group. Others of coronary refer to diagonals, posterior descending branches, posterior branches of the left ventricle, right marginal branch, and so on.

There was a statistically significant difference in QFR results between AF patients and non-AF patients at the average level of all coronary arteries. Then, we sought to distinguish whether these differences were attributed to certain coronary arteries. We compared in the two groups the QFR values of the left anterior descending coronary artery (LAD), left circumflex coronary artery (LCX), right coronary artery (RCA), and other diseased vessels (Figure 4). Results showed a higher QFR ratio in LAD (0.781 ± 0.124 vs. 0.656 ± 0.172, *p* < 0.001), RCA, and other vessels (0.801 ± 0.114 vs. 0.699 ± 0.140, *p* = 0.045). QFR values of LCX showed comparable results (0.814 ± 0.102 vs. 0.751 ± 0.145, *p* = 0.112) between AF patients and non-AF patients.

### 3.4. AF Patients Exhibited Lower Coronary Flow Velocity and Percentage of Stenosis at the Lesion

**Figure 5:**
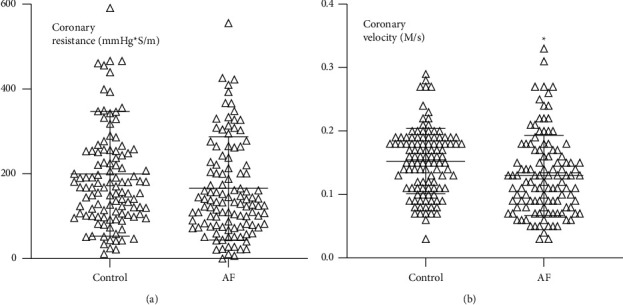
Coronary blood flow resistance (*p*=0.061) and the coronary artery blood flow velocity in the target coronary (*p*=0.003) in AF and non-AF patients.

**Figure 6:**
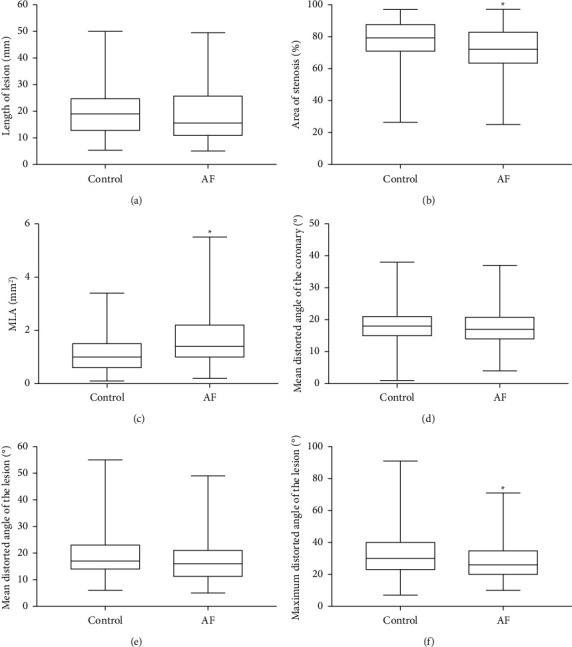
The anatomy of the coronary artery and lesion in the two groups of patients included the length of the lesion, area of stenosis (%), MLA, mean distorted angle in the coronary artery and lesion, and maximum distorted angle in the lesion.

After determining QFR results, we evaluated the relationship between the extent and vascular resistance (mmHg *∗* S/m) of related coronary arteries and the impact of blood flow velocity (M/s) on QFR values in AF patients. There was a lower trend of vascular resistance of the related coronary artery in AF patients than in non-AF patients; however, this difference was not statistically significant (165.9 ± 121.8 vs. 199.9 ± 146.9, *p* = 0.061). It is worth noting that AF patients had lower blood flow velocity than the non-AF patients (0.130 ± 0.063 vs. 0.153 ± 0.052, *p* = 0.003) (Figure 5). These results implied that lower blood flow velocity might be associated with an increased prevalence of QFR values in AF vessels. We further determined a comparable length of lesions (mm) in the two groups of patients (18.83 ± 9.84 vs. 20.11 ± 9.68, *p* = 0.328). Results showed that the area of stenosis (%) at the lesion was significantly lower in AF patients compared to control (71.67 ± 13.66 vs. 77.60 ± 12.47, *p* = 0.001). Consistent with the rate of lumen stenosis, AF patients showed a higher minimum lumen area (MLA, mm^2^) than the control group (1.65 ± 1.03 vs. 1.11 ± 0.65, *p* < 0.001). We then further analyzed the mean distorted angles of coronary arteries (17.47° ± 5.87° vs. 18.63° ± 6.59°, *p* = 0.167) and the lesion around the stenosis (17.36° ± 7.85° vs. 18.71° ± 7.49°, *p* = 0.189), and both showed no significance. However, it is worth noting that AF patients showed a decreased maximum lesion distortion angle compared to the control group (28.14° ± 12.31° vs. 31.95° ± 12.98°, *p* = 0.025) (Figure 6).

### 3.5. Associations of Anatomical Factors and QFR in AF

**Figure 7:**
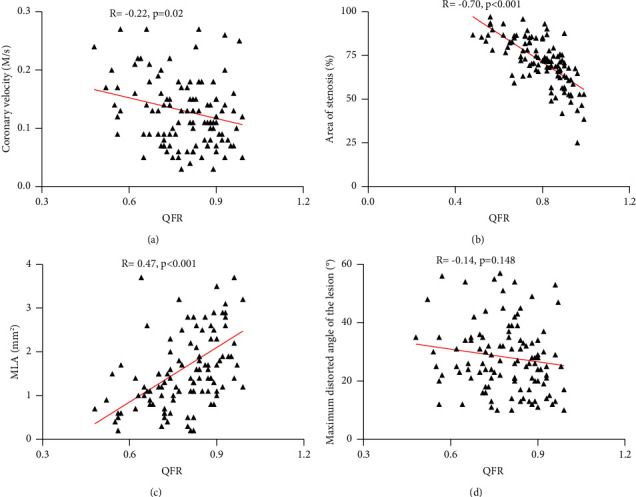
Correlation analysis of coronary flow velocity (*p*=0.02), area of stenosis (%) (*p* < 0.001), MLA (mm^2^) (*p* < 0.001), and maximum distorted angle (*p*=0.148) in the coronary artery with QFR in AF patients.

According to the comparisons of QFR in AF and non-AF patients, we found differences in coronary flow velocity, area of stenosis (%), MLA, and maximum lesion distortion angle. Further correlation analysis showed that both coronary flow velocity (*R* = −0.22, *p*=0.02) and area of stenosis (%) (*R* = −0.70, *p* < 0.001, %) had a negative linear relationship with QFR in AF, whereas MLA presented a positive linear relationship with QFR in AF (*R* = 0.47, *p* < 0.001) (Figure 7). These results suggested that decreased coronary flow velocity and lighter stenosis might lead to higher QFR values in AF patients. The same principle, the larger of MLA and will be the higher of QFR values in these patients.

## 4. Discussion

QFR is an innovative angiographic-based technique using modern software to reconstruct three-dimensional vessels and calculate flow models. This technique has been demonstrated to be superior to angiography-guided PCI as well as medical therapy and also served as a modern, effective, and useable tool. Compared with coronary angiography, QFR has recently enabled interventional cardiologists to determine more easily and accurately whether coronary atherosclerotic plaques are responsible for myocardial ischemia. QFR through computational fluid dynamic analysis has also been demonstrated to be useful in identifying significant stenosis, which correlated with FFR values [10–12].

As is well known that QFR has many advantages as a non-invasive test compared to FFR and is the choice of many interventional physicians, however, the question is why the QFR might be magnified in patients with atrial fibrillation? No one knows. Actually, in patients with AF, the absolute irregularity of the ventricular rate can lead to obvious fluctuation of aortic and coronary pressure. After a long R-R interval, the heartbeat is strong, resulting in higher aortic and coronary pressure. The heartbeat, after a shorter R-R interval, was associated with lower aortic and coronary pressure. There are obvious differences between the two pressure curves. This long-term abnormal rhythm and pressure will lead to the progress of cardiac remodeling. Under continuous cardiac remodeling, the coronary arteries of the AF patients also underwent significant vascular remodeling [13, 14].

Here, at least to some extent, we raised a question of this fact. Whether the QFR result is accurate for a certain group of people, for example, AF patients (it may also be patients with premature contractions or other arrhythmias). Our retrospective study using postinterventional patients to assess whether the use of QFR for guidance is consistent with real-world accuracy is just to highlight the accuracy of QFR in its clinical application for specific populations.

The main objective of the present research was to compare the influence of different blood flow status and anatomical characteristics on QFR results in AF patients. In contrast to non-AF patients, AF patients may have distinct hemodynamic parameters and anatomical features, such as lesion distortion angle [15–17]. Scarsoglio et al. recently proved that a higher ventricular rate during AF exerts an impaired overall coronary blood flow and imbalanced myocardial oxygen supply-demand ratio. The combined increase in the heart rate and higher AF-induced hemodynamic variability led to coronary perfusion impairment [18]. In the present study, we found patients with AF had higher QFR values than non-AF patients. In addition, all of the comparisons including local QFR, residual QFR, and index QFR exhibited significant differences between the two groups. Furthermore, we also found that AF patients showed relatively lower resistance and lower blood flow velocity compared to the non-AF control group.

Despite the excellent correlation and agreement between QFR and FFR, there is discordance of functional ischemia between the two measures [19]. In accordance with our results, previous researchers have reported that physiological characteristics, such as microcirculation, might affect the diagnostic performance of the QFR [20, 21]. Here, we found that AF patients had a lower trend of vascular resistance and lower blood flow velocity in related coronary arteries than non-AF patients. This indicated that the pathological characteristics of coronary microcirculation are different between these two populations. As studies have focused on the mechanisms of AF, fewer researchers have paid attention to hemorheology in AF patients. Recently, Deyranlou et al. proved that AF could alter intracardiac flow and cardiac output that subsequently affects aortic flow circulation [22]. In addition, Keshmiri A determined that AF with a lower flow rate at left ventricular outflow, which in general lowers blood perfusion to systemic and coronary circulations. Consequently, it leads to an endothelial cell activation potential (ECAP) increase and variation of flow structure [23]. Given that, there may be a lack of understanding of such discrepancies and their related factors in AF.

QFR is computationally calculated through three-dimensional reconstruction according to QCA analysis from two different angiographic projections and is therefore directly affected by the visualized definition of target lesion on coronary angiography (CAG) [24, 25], whereas the anterior descending coronary artery is susceptible to overlap of the diagonal or septal branches, and the right coronary artery is susceptible to curvature of the vessels [26, 27]. To eliminate these confounding factors, we next compared corresponding coronary arteries of LAD, RCA, and LCX in the two groups and found that AF patients had a higher QFR ratio in LAD, RCA, and other vessels, while the results in LCX are comparable. In fact, in addition to the three major coronary vessels, there are also PCI for diagonal branches, intermediate branches, and posterior descending branches. We must admit that except for LAD and LCX, there is an obvious difference in the proportion of diseased coronary vessels between the two groups, especially for RCA; as a result, for the balance of the data between the groups, RCA and the other coronary (diagonal branch, intermediate branch, and posterior descending branch) were calculated together. For the QFR of LCX, the result showed a negative statistical difference and we suspect that there may be several reasons: first, the two sets of data are not enough; however, we still found an increased trend of QFR in the AF group. Second, this may ascribe to the fact that we determined the QFR of the circumflex branch from the hepatic position image, and the coronary image at this position is shorter and the vessel diameter is larger.

In addition to the differences in anatomies and microcirculation between the two groups, variances may also be ascribed to baseline heterogeneity of the patients. For example, the AF group had more incidence of stroke and cardiac insufficiency (Table 2), and the AF patients showed decreased eGFR levels compared to the control. These factors may all have contributed to the impairment of the systolic and diastolic capacity of the myocardium. Thereafter, we found that AF patients showed a decreased maximum lesion distortion angle compared to non-AF patients. In addition, we found that both coronary flow velocity and area of stenosis (%) had a negative linear relationship with QFR in AF patients, while MLA presented a positive linear relationship with QFR. Our results suggested that lower coronary flow velocity and lighter stenosis could lead to higher QFR values in AF patients. This offered important groundings for other studies which indicated that a better understanding of these anatomies in AF patients might improve the diagnostic accuracy of QFR analysis [28].

## 5. Conclusion

QFR has enabled interventional cardiologists to determine responsible coronary atherosclerotic plaques for myocardial ischemia more easily and accurately. However, patients with atrial fibrillation have more risk factors as well as specific coronary hemodynamic characteristics. The changes of anatomical structure and blood flow in the coronary arteries of AF patients may increase QFR. It may be ascribed to the decreased blood flow velocity, lighter stenosis, and larger MLA in AF patients. However, in addition to the factors we have determined, we do believe that there should be other unknown factors that might increase QFR values. Better recognition and understanding of these certain anatomies and certain differences in AF patients may assist coronary interventionists to improve their diagnostic accuracy of QFR analysis. Enlarged QFR may result in some AF patients with actual myocardial ischemia not being able to receive reperfusion therapy timely. We encourage cardiologists to be alert to such patients and consider whether these patients have true coronary ischemia from multiple perspectives.

## Figures and Tables

**Table 1:** Baseline characteristics of the patients.

Characteristics	AF patients (*n* = 112)	Control group (*n* = 111)	*p* value
Age (years)	70.28 ± 7.42	68.22 ± 10.53	0.0721
Sex (male), *N*	69 (66.61%)	64 (57.14%)	0.4964
eGFR (mL/min)	67.85 ± 20.26	94.75 ± 29.40	0.0001
Smoke, *N*	39 (34.82%)	36 (32.43%)	0.7058
Heart rate/min	75 ± 13	72 ± 10	0.1318

*Comorbidities*			
Diabetes	39 (34.82%)	44 (39.64%)	0.4567
Hypertension	83 (74.11%)	86 (77.48%)	0.5569
Stroke/TIA	35 (31.25%)	13 (11.71%)	0.0004
NYHA (III-IV)	35 (31.25%)	11 (9.91%)	0.0001

*Serum lipid*			
ox-LDL	2.53 ± 0.74	2.51 ± 0.97	0.8424
TG	1.57 ± 0.96	1.80 ± 0.99	0.0872

Data are expressed as the mean ± standard deviation and *n* (%). NYHA, New York Heart Association classification grading of cardiac function; eGFR, estimated glomerular filtration rate; ox-LDL, oxidized low-density lipoprotein; TG, triacylglycerol.

**Table 2:** Baseline procedural characteristics.

Characteristics	AF patients (*n* = 112)	Control group (*n* = 111)
ACS patients	17 (15.18%)	14 (12.16%)

*Number of drug-eluting stents*		
1	96 (85.71%)	84 (75.68%)
2	16 (14.29%)	26 (23.42%)
≥3	0 (0%)	1 (0.90%)

*PCI vessel*		
LAD	61 (54.46%)	74 (66.67%)
LCX	18 (16.07%)	30 (27.03%)
RCA and other vessels	33 (29.46%)	7 (6.31%)

*Periprocedural treatment*		
Antiplatelet agent	112 (100%)	111 (100%)
GPIIbIIIa	20 (17.86%)	25 (22.52%)
Anticoagulants	109 (97.32%)	20 (18.02%)
*β*-Blocker	95 (84.82%)	97 (87.39%)
Statin	105 (93.75%)	107 (96.40%)
ACEI/ARB/ARNI	59 (52.68%)	48 (43.24%)

Data are expressed as *n* (%). ACS, acute coronary syndrome; GPIIbIIIIa, glycoprotein IIbIIIa receptor blocker; LAD, left anterior descending artery; LCX, left circumflex artery; LM, left main coronary artery; PCI, percutaneous coronary intervention; RCA, right coronary artery. Antiplatelet agent refers to aspirin and clopidogrel or ticagrelor. Anticoagulants refer to warfarin or low molecular heparin or novel oral anticoagulant. ACEI, angiotensin-converting enzyme inhibitor; ARB, angiotensin receptor blocker; ARNI, angiotensin receptor-neprilysin inhibitor.

## Data Availability

All relevant data are available from the corresponding author upon reasonable request.

## Abbreviations

ATP: Adenosine triphosphate
AF: Atrial fibrillation
CAG: Coronary angiography
eGFR: Estimated glomerular filtration rate
FFR: Fractional flow reserve
LAD: The left anterior descending coronary artery
LCX: The left circumflex of the coronary artery
MLA: Minimum lumen area
PCI: Percutaneous transluminal coronary intervention
QFR: The quantitative flow ratio
QCA: Quantitative coronary angiography
RCA: Right coronary artery.

## Supplementary Materials

Supplementary Materials Supplementary Figure 1: we compared LvEF% in baseline data of the two groups and further analyzed the correlation between LvEF% and QRF to determine whether cardiac function directly affects QFR value. Supplementary Figure 2: QFR comparison between the two groups of patients after excluding ACS patients.
