# Supplementary material for: The Differences of Quantitative Flow Ratio in Coronary Artery Stenosis with or without Atrial Fibrillation
Source: J Interv Cardiol. 2023 Oct 13;2023:7278343. doi: 10.1155/2023/7278343 (PMC10589068; doi:10.1155/2023/7278343)
Supplement: Supplementary Materials — Supplementary Figure 1: we compared LvEF% in baseline data of the two groups and further analyzed the correlation between LvEF% and QRF to determine whether cardiac function directly affects QFR value. Supplementary Figure 2: QFR comparison between the two groups of patients after excluding ACS patients. [file 7278343.f1.docx]

**Supplementary Figure 1.**

*

QFR

LVEF (%)

AF

Control

LVEF＜60%

LVEF≥60%

AF

Control

R=0.076, P=0.424

R=0.128, P=0.183

QFR

QFR

LVEF (%)

LVEF (%)

R=0.069, P=0.590

R=0.188, P=0.197

QFR

QFR

LVEF≥60 (%)

LVEF＜60 (%)

**Supplementary Figure 1.** Correlation between cardiac function and QRF. AF patients showed decreased LvEF(%) as compared to the control. QFR did not show difference in patients with normal LvEF(%) and those with declined LvEF% (P=0.980). There was no correlation between LvEF(%) and QFR in either AF patients or the Controls. Neither the subgroup with normal LvEF(%) nor the subgroup with reduced LvEF(%) showed a correlation between LvEF(%) and QFR.

**Supplementary Figure 2.**

QFR of the whole coronary artery excluding ACS

*

Control

AF

**Supplementary Figure 2.** After excluding ACS patients（unstable angina and NSTEMI）, QFR values in the two groups (AF group, n=95; Control group, n=97) were further calculated and the results still showed differences (0.814±0.104 vs.0.705±0.162, p<0.001).
